# Safety from Crime and Physical Activity among Older Adults: A Population-Based Study in Brazil

**DOI:** 10.1155/2012/641010

**Published:** 2012-01-15

**Authors:** Maruí Weber Corseuil, Pedro Curi Hallal, Herton Xavier Corseuil, Ione Jayce Ceola Schneider, Eleonora d'Orsi

**Affiliations:** ^1^Postgraduate Program in Public Health, Federal University of Santa Catarina, 88040-970 Florianopolis, SC, Brazil; ^2^Postgraduate Program in Epidemiology, Federal University of Pelotas, 96010-310 Pelotas, RS, Brazil; ^3^Postgraduate Program in Physical Education, Federal University of Santa Catarina, 88040-970 Florianopolis, SC, Brazil

## Abstract

*Objective*. To evaluate the association between safety from crime and physical activity among older adults. *Methods*. A population-based survey including 1,656 older adults (60+ years) took place in Florianopolis, Brazil, in 2009-2010. Commuting and leisure time physical activity were assessed through the long version of the International Physical Activity Questionnaire. Perception of safety from crime was assessed using the Neighbourhood Environment Walkability Scale. *Results*. Perceiving the neighbourhood as safe during the day was related to a 25% increased likelihood of being active in leisure time (95% CI 1.02–1.53); general perception of safety was also associated with a 25% increase in the likelihood of being active in leisure time (95% CI 1.01–1.54). Street lighting was related to higher levels of commuting physical activity (prevalence ratio: 1.89; 95% CI 1.28–2.80). *Conclusions*. Safety investments are essential for promoting physical activity among older adults in Brazil.

## 1. Introduction

In spite of the well-known benefits of physical activity for health among older adults [1–3], this subgroup of the population is highly susceptible to physical inactivity [4–6]. Therefore, it is increasingly important to understand the correlates and determinants of physical activity behaviours among older adults. Studies have shown that physical activity levels in this age group are influenced by individual factors [4, 6], as well as features of the social and physical environment [7–9].

There is now compelling evidence [7, 10, 11] that environmental characteristics play a role at influencing health-related behaviours, particularly physical activity. Specifically among older adults, for whom the neighbourhood of residence is their predominant context, the aesthetics of the neighbourhood, sidewalks conditions, street lighting, and safety from crime seem to be key correlates of physical activity [12, 13].

Several studies have examined the relationship between safety and physical activity levels; in summary, they suggest that environmental factors may increase our ability to predict physical activity. For example, lack of safety, criminal activities in the neighborhood, inadequate lighting, all of which are not rare in Brazil, have been shown to reduce the likelihood of physical activity, especially among the elderly. [13–17]. On the other hand, perceiving the neighbourhood environment as safe has been shown to encourage older adults to be active in some studies [18–21], but not all of them [22, 23].

The rapid recent urbanization of low and middle income cities has led to concerns related to urban violence. Therefore, understanding the impact of violence on health is a key priority for public health. It is known that feeling unsafe generates fear and insecurity among community members, which could end up limiting mobility in the neighbourhood [24]. The aim of this study was to evaluate the association between perception of safety from crime and physical activity among older adults living in a southern Brazilian city.

## 2. Methods

### 2.1. Population and Study Design

A population-based cross-sectional study was conducted from September, 2009 to June, 2010 in the city of Florianopolis, Brazil. The sample included individuals aged 60 years or more. This study is part of a broader survey on health of older adults. ^1^ Florianopolis is the capital of the state of Santa Catarina, in South Brazil. Its population comprises just over 400,000 inhabitants, of which 10% are aged 60 years or more. ^2^ In the most recent national census, Florianopolis presented a *per capita *income of R$ 701.4 (equivalent to US$ 350), a Human Development Index of 0.88, the highest among Brazilian state capitals, a Gini index for income inequality of 0.57, and life expectancy at birth of 72.8 years. ^3^

A multistage sampling strategy was used. In Brazil, all cities are divided into census tracts (delimited areas comprising around 300 households each). The 420 census tracts of the city were the primary sampling units in our survey. They were categorized into 10 groups according to its mean income level. We sampled 8 tracts in each stratum, totalling 80 census tracts in the sample. Within each sampled tract, households were selected randomly, and every older adult living in each selected household was interviewed. In cases where no eligible older adults were living in the sampled household, the next one (to the right) was selected. Elderly people with severe cognitive problems had the questionnaire completed by caregivers/guardians. Institutionalized subjects were not included.

In each sampled household, interviewers tried to interview participants at least four times before considering them as losses. At least one of these attempts took place in the evening and one in a weekend day. Subjects were allowed to refuse to take part in the survey. No replacements were done. Interviewers were trained for 40 hours in the administration and coding of the questionnaire prior to data collection. The study protocol was approved by the Federal University of Santa Catarina Ethics Committee (protocol 352/2008) on the 23rd of December, 2008.

### 2.2. Measures

The long version of the International Physical Activity Questionnaire (IPAQ), adapted and validated in Brazil [25], was used. As recommended, we included in this analysis only the leisure time and commuting sections of IPAQ [26]. Commuting physical activity was expressed as the sum of minutes per week spent walking and cycling for transportation. Leisure time physical activity was also expressed in minutes per week, as follows: (minutes per week of walking for leisure) + (minutes per week on moderate-intensity physical activity) + (minutes per week on vigorous-intensity physical activity ∗ 2). A cutoff of 150 minutes per week was employed [5, 27]. 

Independent variables included the questions about safety from crime from the Neighbourhood Environmental Walkability Scale (NEWS), validated in Brazil [28–30]. Response choices were dichotomized in the “yes/no” format, as described previously [29, 30]. The questions included in the present analyses were as follows. (1) Are the streets near your household well illuminated at night? (2) Do you feel it is safe to walk, cycle or practice sports in your neighbourhood during the day? (3) Do you feel it is safe to walk, cycle or practice sports in your neighbourhood at night? We also constructed a combined score ranging from zero (subjects who answered “no” to all three questions) to three (subjects who answered “yes” to all three questions).

Other covariates included sex, age (60–69, 70–79, and 80+ years), skin colour (white, black, and mixed), schooling level (0–8, 9–11, and 12+ years of education), per capita family income (divided into tertiles: 1° tertile ≤ R$ 450,00; 2° tertile > R$ 450,00 ≤ R$ 1.130,00; 3° tertile > R$ 1.130,00), self-rated health (excellent/good, fair, and poor/very poor), disability score for basic and instrumental daily activities (no disability, mild disability—needs help to perform 1–3 activities, and moderate/severe disability—needs help to perform 4 or more activities) [31].

### 2.3. Data Analysis

In the unadjusted analysis, we compared the proportion of older adults in each physical activity category according to perceptions of safety through Chi-square tests. In order to test the adjusted association between safety from crime and physical activity, we ran Poisson regression models with robust adjustment for the variance [32]. Significance levels were calculated using Wald tests. Analyses were run using Stata 9.0 (Stata Corp., College Station, Estados Unidos). All analyses took the clustering of the sample into account by using the set of commands “svy” available in Stata.

## 3. Results

Within the sampled households, we located 1,911 older adults who were eligible to take part in our survey. Of them, we were able to interview 1,705 (89.2%). We had 49 interviews with carers (for subjects with severe cognitive impairment) and opted to exclude them from all analyses, because our variables of interest deal with subject perceptions of environmental factors.

The sample included 63.9% of women, with an age range of 60 to 102 years (mean 70.4, SD 7.8). Almost 2/3 of the older adults had eight years or less of schooling. The average per capita income was R$ 1.347,98 (SD R$ 2.596,28). In terms of disability for basic and instrumental daily life activities, most individuals reported mild-to-moderate dependency. Over half of the participants rated his/her health as excellent/good (Table 1).

The proportion of older adults reaching 150 minutes per week of leisure time physical activity was 29.6% (95% CI 27.4–32.0); this proportion was higher among men (35.6%; 95% CI 31.8–39.5) as compared to women (26.3%; 95% CI 23.6–28.9). In total, 28.0% (95% CI 25.8–30.1) of the participants reported 150 minutes per week or more of commuting physical activity; this proportion was higher in men (36.1%; 95% CI 32.3–40.0) as compared to women (23.4%; 95% CI 20.8–25.9). Mean minutes per week of leisure time physical activity was 131.8 (SD 216); it was 123.6 minutes per week (SD 197.5) commuting physical activity.

Table 2 shows the prevalence of leisure time and commuting physical activity according to the perception of safety variables.

In the unadjusted analyses, feeling safe to walk, cycle, or practice sports in the neighbourhood during the day and the score of safety were associated with leisure time physical activity (Table 3). In terms of commuting physical activity, street lighting was a significant correlate in the unadjusted analyses (Table 4).

The adjusted analyses (Tables 3 and 4) confirmed the results of the unadjusted one. Perceiving the neighbourhood as safe during the day was related to a 25% increased likelihood of being active in leisure time (95% CI 1.02–1.53); general perception of safety was also associated with a 25% increase in the likelihood of being active in leisure time (95% CI 1.01–1.54). Street lighting was related to higher levels of commuting physical activity (prevalence ratio: 1.89; 95% CI 1.28–2.80).

## 4. Discussion

In summary, feeling safe was related to higher levels of physical activity among older adults living in Florianopolis, Brazil. This finding is particularly relevant in the context of low- and middle-income countries, where safety concerns are growing with rapid urbanization.

Our findings are in accordance with a previous study, which reported that elderly men who feel safe to walk, cycle, or practice sports in the neighbourhood during the day are more likely to be active in their leisure time, as compared to their counterparts who do not feel safe to do so [29]. In the US, older adults who reported feeling safe were 29% more likely to be active in their leisure time than the others, even after adjusting for confounding factors [33]. Similarly, persons who perceived their neighborhood as less than “extremely safe” were more than twice as likely to be inactive in leisure time as compared to those who felt the neighborhood as “extremely safe.” Furthermore, those who considered it to be “not at all safe” were nearly three times more likely to have no leisure time physical activity as compared to the reference group [16]. Also, women who reported low crime in their neighbourhoods reported engaging in more moderate and vigorous physical activity per week than those with higher crime rates in the neighborhood. Interestingly, the study also suggests that older adults may be more susceptible to environmental variables than younger people [34].

Taken together, this evidence suggests that interventions to promote physical activity are likely to fail if they focus exclusively on individual and social factors. Places should also be the target of interventions so that environments become increasingly conductive to physical activity and other healthy choices [35].

Commuting physical activity was also related to the perception of safety. Street lighting, for example, was directly associated with physical activity. Florindo and coworkers [36] found the same association we detected in a sample of adults living in Sao Paulo. In a population-based study in England, it was found that women were 47% less likely to report walking for at least 15 minutes per week if they reported feeling unsafe to walk in their neighborhood during the day as compared with women who felt safer. [37]. In the US, study showed that areas reported by the elders that were safe for walking were significantly related to walking activity [15]. Other study in high income countries have reported similar associations [12].

Previous reports also showed that neighbourhood safety problems are related to functional disability among older adults, an association that is likely mediated by reduced physical activity levels [38].

As with most studies on the association between physical activity and the environment, the cross-sectional nature of our data impedes us from discussing causality. Longitudinal studies are particularly needed in this area. We did not collect objective information about safety (e.g., neighbourhood crime rates), but specifically for the association described in this paper, we assume features of the perceived environment are as useful as (if not more than) objective measures. Another potential limitation, that is inherent to observational studies, is that it is possible that we did not collect information on some relevant confounders. For less than 3% of the older adults sampled, information was collected via caregivers; these subjects were excluded from the analyses due to the subjective nature of the variables of interest. There is evidence that individuals tend to overreport physical activity when answering to IPAQ [39]. Also, IPAQ is recommended for adults up to the age of 64 years only. However, several studies in Latin America have confirmed the feasibility of administering IPAQ to older adults [25, 26, 29]. In spite of all potential limitations of the questionnaire, there is no reason to believe that bias would differ across categories of the environmental variables, and therefore, our results are unlikely to be affected by limitations of the questionnaire. Finally, although we asked separate questions about safety from traffic, it is still possible that some respondents considered both safety from crime and safety from traffic when answering to the questions analyzed in this paper.

Some strengths of our study should also be highlighted. Very few studies have explored the association between safety and physical activity in low- and middle-income countries; in addition, analyzing this association in a sample of older adults is rare. We obtained high response rates, which strengthens the internal validity of our findings. The uniform distribution of the losses in the family income deciles contributed to this condition of sample inference. Our sample is similar to the whole city's population in terms of sociodemographic distribution,^4^ thus reinforcing the random nature of our sampling strategy. However, because our sample was drawn from only one state capital in Brazil, which happens to be the one with the highest HDI, extrapolating our findings to Brazil as a whole may be misleading. We used Poisson instead of logistic regression due to the high prevalence of the outcomes under investigation, as suggested in a previous publication [32]. Another strength of our design was to evaluate separately leisure time and commuting physical activity, which have been shown to present different correlates in other Brazilian studies [29, 30].

 Brazilian cities need to invest on improving safety for several reasons, including reducing the burden of accidents and violence, which are currently key determinants of morbidity and mortality in the country. Our study shows that such investments, at least in Florianopolis, may also generate benefits for the health of the population, by increasing physical activity levels of older adults. This is particularly relevant for low-income older adults, who are unable to use other modes of transportation and are therefore more likely to benefit from improved safety in their neighbourhoods.

## Figures and Tables

**Table 1:** Description of the sample according to demographic, socioeconomic, and health conditions in older adults. Florianopolis, South Brazil, 2009/2010.

Variables	*n* (%)	Leisure time physical activity		Commuting physical activity	
		≥150 min/wk (%)	P**	≥150 min/wk (%)	P**
Gender			<0.001		<0.001
Females	1058 (63.9)	26.3		23.4	
Males	598 (36.1)	35.6		36.1	
Age (years)			<0.001		<0.001
60 to 69	846 (51.1)	33.7		31.7	
70 to 79	596 (36.0)	28.5		27.2	
80 or more	214 (12.9)	16.2		14.2	
Skin color			0.46		0.80
White	1410 (86.8)	30.3		28.1	
Mixed	131 (8.0)	25.2		29.0	
Black	84 (5.2)	28.6		25.0	
Marital status			<0.001		<0.001
Married	974 (58.8)	32.0		29.5	
Single, separated, or divorced	225 (13.6)	33.8		35.1	
Widowed	457 (27.6)	22.5		21.2	
Per capita monthly income			<0.001		0.01
Lower tertile	552 (33.3)	23.2		25.7	
Middle tertile	550 (33.2)	27.5		25.5	
Highest tertile	554 (33.5)	38.3		35.7	
Schooling (years)			<0.001		<0.001
0 to 8	1031 (62.6)	23.1		24.4	
9 to11	231 (14.0)	35.1		39.4	
12 or more	386 (23.4)	44.3		30.8	
Disability score*			<0.001		<0.001
No disability	458 (27.7)	39.5		38.9	
Mild disability	707 (42.7)	31.8		29.7	
Moderate/severe disability	491 (29.6)	17.3		15.3	
Self-reported health status			<0.001		<0.001
Excellent/good	848 (51.2)	37.4		33.1	
Fair	642 (38.8)	22.3		24.1	
Poor/very poor	165 (10.0)	18.8		16.4	

Notes: *Measured by the activities of daily living scale.

**P value of Chi-square test.

**Table 2:** Prevalence of physical activity according to perception of safety in older adults. Florianopolis, South Brazil, 2009/2010.

Variables	*n*	Leisure time physical activity			Commuting physical activity		
		≥150 min/wk (%)	<150 min/wk (%)	P*	≥150 min/wk (%)	<150 min/wk (%)	P*
Street lighting at night				0.82			0.01
Yes	1490	29.8	70.2		29.0	71.0	
No	159	28.9	71.1		19.5	80.5	
Safe to walk during the day				0.02			0.29
Yes	1273	31.3	68.7		28.7	71.3	
No	370	25.1	74.9		25.9	74.1	
Safe to walk during the night				0.69			0.32
Yes	539	29.3	70.7		26.7	73.3	
No	1091	30.3	69.7		29.1	70.9	
Perception of safety score				0.008			0.15
Good	1198	31.8	68.2		29.3	70.7	
Poor	428	25.0	75.0		25.7	74.3	

Notes: *P value of Chi-square test.

**Table 3:** Table Unadjusted and adjusted associations between perception of safety and leisure-time physical activity in older adults. Florianopolis, South Brazil, 2009/2010.

Variables	Unadjusted model			Adjusted model		
	PR	95% CI	*P*	PR	95% CI	*P*
Street lighting at night^a^			0.61			0.91
No	1.00			1.00		
Yes	1.09	0.78; 1.53		0.98	0.71; 1.37	
Safe to walk during the day^a^			0.02			0.03
No	1.00			1.00		
Yes	1.28	1.04; 1.58		1.25	1.02; 1.53	
Safe to walk during the night^a^			0.94			0.51
No	1.00			1.00		
Yes	0.99	0.78; 1.26		0.93	0.75; 1.16	
Perception of safety score^b^			0.01			0.03
Poor	1.00			1.00		
Good	1.32	1.07; 1.64		1.25	1.01; 1.54	

^a^Multiple analysis consists of the variables street lighting at night, safe to walk during the day, and safe to walk during the night, adjusted for sex, age, schooling, disability score, and self-perceived health.

^b^Multiple analysis consists of the variable perception of safety score adjusted for sex, age, schooling, disability score, and self-perceived health.

**Table 4:** Unadjusted and adjusted associations between perception of safety and commuting physical activity in older adults. Florianopolis, South Brazil, 2009/2010.

Variables	Unadjusted model			Adjusted model		
	PR	95% CI	*P*	PR	95% CI	*P*
Street lighting at night^a^			0.001			0.002
No	1.00			1.00		
Yes	1.97	1.33; 1.92		1.89	1.28; 2.80	
Safe to walk during the day^a^			0.86			0.97
No	1.00			1.00		
Yes	1.03	0.77; 1.37		1.00	0.75; 1.32	
Safe to walk during the night^a^			0.46			0.05
No	1.00			1.00		
Yes	0.94	0.78; 1.12		0.85	0.71; 1.01	
Perception of safety score^b^			0.48			0.85
Poor	1.00			1.00		
Good	1.10	0.83; 1.46		1.03	0.78; 1.35	

^a^Multiple analysis consists of the variables street lighting at night, safe to walk during the day, and safe to walk during the night, adjusted for sex, age, schooling, disability score, and self-perceived health.

^b^Multiple analysis consists of the variable perception of safety score adjusted for sex, age, schooling, disability score, and self-perceived health.

## Endnotes

1. Pesquisa “Condições de saúde da população idosa do município de Florianópolis-SC: estudo de base populacional” financiada pelo Conselho nacional de Desenvolvimento Científico e Tecnológico (CNPq).
2. IBGE-Instituto Brasileiro de Geografia e Estatística. Estimativas populacionais para o TCU. Estimativas da população para 1° de julho de 2009. (http://www.ibge.gov.br/home/estatistica/populacao/estimativa2009/POP2009_DOU.pdf).
3. Programa das Nações Unidas para o Desenvolvimento. Atlas do Desenvolvimento Humano no Brasil, 2003. (http://www.pnud.org.br/atlas/).
4. IBGE-Instituto Brasileiro de Geografia e Estatística, Profile of Elderly Heads of Household in Brazil 2000, Rio de Janeiro, 2002.
